# Emergence of linezolid- and vancomycin-resistant *Enterococcus faecium* in a department for hematologic stem cell transplantation

**DOI:** 10.1186/s13756-016-0131-6

**Published:** 2016-09-22

**Authors:** M. Krull, I. Klare, B. Ross, R. Trenschel, D. W. Beelen, D. Todt, E. Steinmann, J. Buer, P-M. Rath, J. Steinmann

**Affiliations:** 1 Hospital Hygiene, University Hospital Essen, University Duisburg-Essen, Essen, Germany; 2 Wernigerode Branch, Robert Koch Institute, Wernigerode, Germany; 3 Department of Bone Marrow Transplantation (AHE), West German Cancer Center, University Hospital Essen, Essen, Germany; 4 TWINCORE Centre for Experimental and Clinical Infection Research; a joint venture between the Medical School Hannover (MHH) and the Helmholtz Centre for Infection Research (HZI), Institute for Experimental Virology, Hannover, Germany; 5 Institute of Medical Microbiology, University Hospital Essen, University Duisburg-Essen, Hufelandstr. 55, 45122 Essen, Germany

**Keywords:** VRE, Linezolid-resistant VRE, Hematologic stem cell transplantation, Genotyping by PFGE

## Abstract

**Background:**

Prevalence of vancomycin-resistant enterococci has increased in Germany. Here, we report the cluster of linezolid- and vancomycin-resistant *Enterococcus faecium* (LVRE) in a German department for hematologic stem cell transplantation (HSCT).

**Methods:**

In this retrospective analysis we included all patients with LVRE in a university-based department for HSCT in 2014 and 2015. Patients chart reviews were used to investigate the epidemiology and clinical outcome. Available LVRE isolates underwent detailed microbiological characterization and genotyping by pulsed-field gel electrophoresis (PFGE).

**Results:**

In total, 20 patients with LVRE were identified within the observed time period. All except two patients underwent allogeneic HSCT. Surveillance culture results from incoming patients and chart review revealed that 10 of 20 patients were colonized at hospital admission. Eight of 10 patients with in-hospital acquired LVRE had previous linezolid treatment. Analysis of spatio-temporal patterns showed no evidence for LVRE patient-to-patient or environment-to-patient transmission within the HSCT department. In five cases (25 %) LVRE bloodstream infection occurred. Nine LVRE isolates could be saved for characterization. Eight isolates carried *vanA*, one isolate *vanB*. PFGE analysis showed that four different LVRE clones were responsible for the cluster. One single genotype was present in six LVRE isolates whereupon the corresponding patients were all referred from the same hospital to the HSCT department.

**Conclusions:**

This is the first report demonstrating the emergence of LVRE in a German HSCT department. (L)VRE screening at patients’ admission and appropriate infection control strategies were sufficient to prevent any transmission. Further studies in this predisposed patient collective are warranted.

## Background

Data from 2014 of the European Antimicrobial Resistance Surveillance Network (EARS-Net) reported the proportion of vancomycin resistance in *Enterococcus faecium* in Europe being 8.9 % [1]. The VRE rate in Germany has increased dramatically in the last years [2]: Between 2007 and 2012 surgical site and bloodstream infections due to VRE increased significantly (526 and 278 %) [2].

Infections with VRE are a major cause of morbidity in HSCT recipients [3]. Universal use of central venous catheters, heavy exposure to prophylactic and therapeutic anti-infectives, numerous periods of hospitalization and extensive contact to health-care providers predisposes this patient cohort to colonization and subsequent infections with resistant pathogens such as VRE [4].

Linezolid resistance in VRE (LVRE) is an uncommon finding. Case reports and outbreaks of LVRE have been described from US, Greece, Italy and Ireland mainly in intensive care unit patients, patients after solid organ transplantation or with hematological malignancy [5–10]. Linezolid exposure and patient-to-patient transmission were shown to be main factors for LVRE infection [11].

In this retrospective study (2014–2015) cases with LVRE in a university-based HSCT department were reviewed to understand the epidemiology. In addition, LVRE isolates were characterized and genotyped to assess possible strain transmission between patients.

## Methods

### Setting and patient population

The epidemiology of linezolid resistance in VRE was investigated in the department for HSCT belonging to the West German Cancer Center within the University Hospital Essen over a period of 2 years (2014–2015). The HSCT department provides specialist superregional services for adult patients with hematological malignancies. In 2014 and 2015 394 patients underwent allogeneic HSCT. The clinic consists of three spatially separated wards. The VRE rate (one isolate per patient) was in 2014 9.6 % and in 2015 15 %. All patients positive for LVRE and with a stay in the HSCT department in the year 2014 and 2015 were included in the study. Patient chart review was performed to retrieve clinical information from the patients with LVRE.

### Infection control measures

All incoming patients were screened for VRE by obtaining rectal swabs or stool samples in outpatient facilities before admission to the HSCT department. From every hospitalized HSCT patient, surveillance cultures (rectal swab/stool sample, urine, blood, respiratory secretions) were weekly obtained. Infection control measures were performed according to guidelines for preventing infectious complications among hematopoietic cell transplant recipients: All patients were placed in single-patient rooms. The rooms are protective environment rooms consisting with high efficiency particulate air filter (HEPA). Standard precautions include hand hygiene and wearing personal protective equipment (gown, cap and gloves) were recommended for hospital staff and visitors before entering the room [12]. Environmental samples from patient’s rooms (sink, shower etc.) were investigated on a regular basis (every 4 weeks in each patient’s room) for bacterial and fungal contamination. The swabs were cultured on standard media (blood agar, MacConkey agar, chromogenic Candida agar).

### Bacterial isolation, identification and antibiotic-susceptibility testing

Chromogenic medium (VRESelect, Bio-Rad, Munich, Germany) selective for VRE was used for all rectal and stool screening samples. Species identification was performed by using MALDI-TOF mass spectrometry (VITEK MS, bioMérieux, Nürtingen, Germany). Susceptibility testing was conducted using the VITEK 2 system (bioMérieux) and interpretation of clinical MIC breakpoints was performed according to EUCAST. The MIC breakpoint of linezolid according to EUCAST is > 4 μg/mL . For daptomycin the breakpoint (>4 μg/ml) was used from CLSI. All isolates with resistance against linezolid were confirmed by linezolid MIC test strip (Liofilchem, Roseto degli Abruzzi, Italy). Saved isolates were stored at −80 °C until further characterization.

In total, nine isolates were available for further analyses at the German Reference Center for Staphylococci and Enterococci (Robert Koch Institute, Wernigerode, Germany). There, antibiotic susceptibility testing was performed by broth microdilution (BMD) as previously described [13].

### Determination of van, virulence and cfr genes

PCR analysis for glycopeptide resistance genes (*vanA* and *vanB*) and virulence markers (*esp*, *hyl*) was performed using standard protocols [14]. Part of the *cfr* gene was amplified as previously described [15]. Nucleotide changes in 23S rDNA conferring linezolid resistance were determined and calculated using a previously described assay. It is a combination of a PCR amplification of the six 23S rDNA alleles in *E. faecium* followed by a restriction digestion recognizing the mutated nucleotide position and a subsequent gelchip-based separation of the digested vs. non-digested fragments allowing a quantification [16].

### Macrorestriction analysis

Macrorestriction analysis for the LVRE isolates were performed using the restriction endonuclease *Sma*I with subsequent PFGE as reported [13].

### Statistics

Graphics were generated with GraphPad Prism (La Jolla, USA).

## Results

### Characteristics of LVRE patients

In the observed time period, 20 patients were found to be colonized or infected with LVRE in the HSCT department. The percentage of LVRE among VRE in the HSCT department was 27 % in 2014 and 20 % in 2015. All patient characteristics are shown in Table 1. Eighteen of these 20 patients underwent HSCT; the two remaining patients had a hematological malignancy with prolonged neutropenia. In five cases (25 %) a bloodstream infection with LVRE occurred. In two of the five cases (40 %) the LVRE bacteremia was associated with death of the patient. In the other 15 cases, LVRE was predominantly found in the gut (positive stool or rectal swab) or in the urinary tract. Overall, eight patients died on the HSCT ward or were discharged into palliative care (40 %).

**Table 1:** Clinical characteristics of 20 patients with linezolid- and vancomycin-resistant *Enterococcus faecium* (LVRE) and phenotypic and genotypic features of nine further characterized LVRE isolates

Patient no.	Sex (age [yr])	Underlying Disease	Allogeneic HSCT	Localisation of LVRE (except bloodstream)	Bloodstream infection with LVRE	Previous linezolid exoposure	Clinical outcome	Previous hospital	MICs (mg/L) of the nine further characterized LVRE isolate				PFGE type
									Linezolid	Vancomycin	Teicoplanin	Daptomycin	
1	M (66)	lymphoma	yes	rectal	no	yes	death	A	–	–	–	–	–
2	M (64)	arthritis-metothrexat	no	urine	no	yes	discharge	unknown	–	–	–	–	–
3	F (34)	acute myeloic leukemia	yes	rectal, inguinal	yes	yes	death	C	–	–	–	–	–
4	F (47)	acute myeloic leukemia	yes	rectal, urine	no	unknwon	discharge	D	–	–	–	–	–
5	M (70)	acute myeloic leukemia	yes	rectal	yes	yes	discharge	E	–	–	–	–	–
6	F (54)	acute myeloic leukemia	yes	rectal, oral cavity, BAL	no	unknown	death	D	–	–	–	–	–
7	F (48)	myelodysplastic syndrome	yes	rectal, inguinal, wound	yes	yes	death	C	16	512	64	2	II
8	F (67)	acute lymphatic leukemia	yes	anal, inguinal, oral cavity	yes	unknown	discharge in palliative care	D	–	–	–	–	–
9	F (70)	myelodysplastic syndrome	yes	rectal, armpit, urine	no	yes	discharge	D	>32	512	128	2	I
10	F (68)	aplastic anemia	yes	urine	no	unknown	death	D	–	–	–	–	–
11	F (20)	acute myeloic leukemia	yes	urine	no	unknown	discharge	D	–	–	–	–	–
12	M (58)	myelodysplastic syndrome	yes	rectal, inguinal	no	unknown	discharge	D	32	512	128	2	I
13	F (58)	acute myeloic leukemia	yes	rectal, urine	no	unknown	discharge	D	16	512	128	2	I
14	M (53)	acute myeloic leukemia	yes	rectal, hair	yes	no	discharge	D	16	512	128	4	I
15	F (10)	histiocytosis	yes	rectal, urine	no	unknown	discharge	F	–	–	–	–	–
16	F (22)	acute myeloic leukemia	yes	rectal	no	no	discharge	D	16	512	128	2	I
17	M (62)	acute myeloic leukemia	yes	rectal, oral cavity	no	unknown	discharge	D	16	512	128	2	I
18	M (57)	acute myeloic leukemia	yes	rectal, urine	no	unknown	death	D	–	–	–	–	–
19	F (70)	acute myeloic leukemia	yes	rectal, oral cavity, urine	no	yes	discharge	G	32	512	128	2	IV
20	M (54)	multiple myeloma	no	rectal	no	yes	Discharge in palliative care	H	16	1024	<1	2	III

*Abbreviations*: *MIC* minimum inhibitory concentration, *PFGE* pulsed-field gel electrophoresis

### Epidemiological investigations

**Fig. 1:**
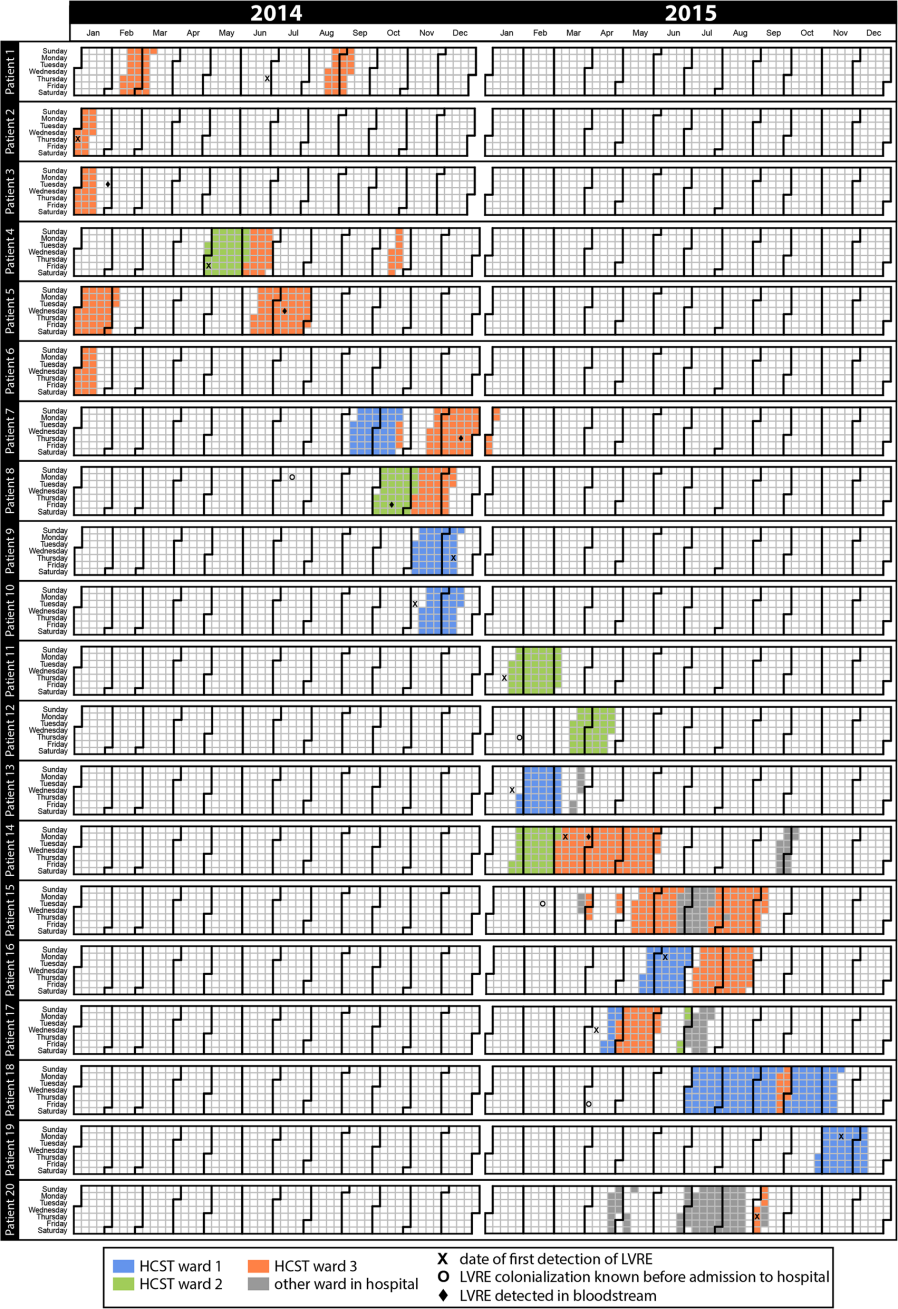
Epidemiological map of 20 patients with LVRE on three wards in the department of hematologic stem cell transplantation. For patient number 6 the first detection of LVRE was already at the end of 2013. A stay in ambulance is only presented, if there was a detection of LVRE

Chart review revealed that all three spatially separated wards were affected (Fig. 1). All LVRE patients stayed in different patient rooms. Results from routine microbiological surveillance swabs from the patient-near environment exhibit no LVRE (data not shown).

In 10 of 20 patients LVRE was found before or at date of admission in the HSCT department. By analysis of the hospitals from which the patients were referred, 12 of 20 patients were previously treated in hospital D (Table 1). Based on the epidemiological analysis there was no evidence for LVRE transmissions within the HSCT department (Fig. 1). Pretreatment with linezolid before LVRE detection could only be demonstrated for the patients with LVRE acquisition during the hospital stay. Eight of 10 patients had prior linezolid therapy before LVRE detection (Table 1).

### Antimicrobial susceptibility profiles and molecular characterization of LVRE

All 20 LVRE isolates had MICs for linezolid of ≥ 16 mg/L. The in vitro activity using BMD method for linezolid, vancomycin, teicoplanin and daptomycin against the nine saved LVRE isolates are shown in Table 1. All tested isolates were positive for *esp* and two for *hyl*. Eight LVRE carried *vanA*, one isolate *vanB* (patient number 20). PCR for *cfr* gene was negative for all isolates. The most frequent resistance mechanism in *Enterococcus* is a nucleotide change at position 2576 (G2576U) in the central region of domain V of 23S rRNA. All nine linezolid-resistant isolates possessed a combination of wildtype and mutated allele variants typical for clinical enterococcal isolates showing linezolid resistance.

### PFGE typing

PFGE analysis of 9 LVRE isolates showed in total four unrelated genotypes. In six isolates, one single clone could be detected. These isolates belong to patients that were treated in hospital D before admission to the HSCT department. The three other LVRE were not closely genetically related.

## Discussion

To the best of our knowledge, this report constitutes the first description of a cluster of LVRE in HSCT recipients and shows also the first epidemiological data from Germany.

One study reported that immunodeficiency, hematological malignancy, hospitalization in a hematological ward and prolonged antibiotic treatment have been identified as risk factors for the development of linezolid resistance in enterococci [7]. Furthermore, pre-treatment with linezolid was shown to select for LVRE [17]. However, LVRE can also be found in patients without previous linezolid therapy [18].

LVRE infections are a serious therapeutic challenge as alternative antimicrobial agents are strongly limited. Twenty-five percent of HSCT recipients had LVRE bacteremia and 40 % of these were associated with fatal outcome. Daptomycin as a new last resort antibiotic can be in these cases one feasible option. According to the MIC-breakpoint of CLSI for daptomycin (≤4 mg/L) all nine LVRE isolates were susceptible to this antibiotic. It was recently reported that *Enterococcus faecium* bacteremia with increased daptomycin MICs (3–4 μg/mL) was shown to be associated with predicted microbiological failure of daptomycin therapy [19]. One of the isolates had a daptomycin MIC of 4 mg/L.

The majority of our HSCT patients had multiple risk factors for colonization and/or infection with (L)VRE. Based on the epidemiological analyses and the VRE screening results at admission we found that 60 % of LVRE patients were referred from one specific hospital (D) from which two-thirds had LVRE at the date of admission to the HSCT unit. Furthermore, the typing analyses showed that six of the nine saved LVRE isolates were clonally related. The patients with these clones were all pretreated in hospital D indicating that this clonal cluster was brought into the HSCT department from an external source.

In contrast, the identification of three other unrelated LVRE genotypes from different patients underscores that the cluster of LVRE was not only due to this “local outbreak in hospital D” but was rather a multifactorial event of LVRE emergence in a high-risk patient collective. On the one hand, LVRE emerged due to linezolid therapy which received 8 of 10 patients without LVRE at admission. On the other hand, there were also independent events of de novo selection of linezolid-resistant mutants in VRE-positive patients.

Even though, the HSCT department was heavily affected with LVRE patients we found no hints for transmission between patients or from the environment to the patients. The surveillance screening of all incoming patients for (L)VRE for the early detection and prompt initiation of hygienic barrier measures for all affected patients may be the reason why there is no proof for LVRE patient-to-patient transmission within the HSCT department. Our study has some limitations. Firstly, it was not possible to save all LVRE isolates of the affected patients. Thus, the expressiveness concerning the clonal spread is limited. Secondly, due to the retrospective study design we were not able to give enough evidence for certain risk factors for LVRE colonization/infection or treatment outcome in our HSCT recipients. Also, this is a case only study that has no comparison group. Further studies to identify risk factors and to determine optimal treatment in this predisposed high-risk patient collective are warranted.

## Conclusions

In conclusion, LVRE emerged in HSCT recipients in a German university hospital. Our study indicates that (L)VRE screening at patients’ admission and appropriate infection control strategies were sufficient to prevent any intra-department transmissions.

## Abbreviations

HSCT: Hematologic stem cell transplantation
LVRE: Linezolid- and vancomycin-resistant enterococci
PFGE: Pulsed-field gel electrophoresis
VRE: Vancomycin-resistant enterococci
